# Hidden genetics behind glomerular scars: an opportunity to understand the heterogeneity of focal segmental glomerulosclerosis?

**DOI:** 10.1007/s00467-023-06046-1

**Published:** 2023-09-20

**Authors:** Adele Mitrotti, Marica Giliberti, Vincenzo Di Leo, Ighli di Bari, Paola Pontrelli, Loreto Gesualdo

**Affiliations:** Precision and Regenerative Medicine and Ionian Area, Nephrology, Dialysis and Transplantation Unit, Department of Emergency and Organ Transplantation, University of Bari Aldo Moro, Bari, Italy

**Keywords:** Focal segmental glomerulosclerosis, Nephrotic syndrome, Glomerular scars, Genetics

## Abstract

Focal segmental glomerulosclerosis (FSGS) is a complex disease which describes different kinds of kidney defects, not exclusively linked with podocyte defects. Since nephrin mutation was first described in association with early-onset nephrotic syndrome (NS), many advancements have been made in understanding genetic patterns associated with FSGS. New genetic causes of FSGS have been discovered, displaying unexpected genotypes, and recognizing possible site of damage. Many recent large-scale sequencing analyses on patients affected by idiopathic chronic kidney disease (CKD), kidney failure (KF) of unknown origin, or classified as FSGS, have revealed collagen alpha IV genes, as one of the most frequent sites of pathogenic mutations. Also, recent interest in complex and systemic lysosomal storage diseases, such as Fabry disease, has highlighted *GLA* mutations as possible causes of FSGS. Tubulointerstitial disease, recently classified by KDIGO based on genetic subtypes, when associated with *UMOD* variants, may phenotypically gain FSGS features, as well as ciliopathy genes or others, otherwise leading to completely different phenotypes, but found carrying pathogenic variants with associated FSGS phenotype. Thus, glomerulosclerosis may conceal different heterogeneous conditions. When a kidney biopsy is performed, the principal objective is to provide an accurate diagnosis. The broad spectrum of phenotypic expression and genetic complexity is demonstrating that a combined path of management needs to be applied. Genetic investigation should not be reserved only to selected cases, but rather part of medical management, integrating with clinical and renal pathology records. FSGS heterogeneity should be interpreted as an interesting opportunity to discover new pathways of CKD, requiring prompt genotype–phenotype correlation. In this review, we aim to highlight how FSGS represents a peculiar kidney condition, demanding multidisciplinary management, and in which genetic analysis may solve some otherwise unrevealed idiopathic cases. Unfortunately there is not a uniform correlation between specific mutations and FSGS morphological classes, as the same variants may be identified in familial cases or sporadic FSGS/NS or manifest a variable spectrum of the same disease. These non-specific features make diagnosis challenging. The complexity of FSGS genotypes requires new directions. Old morphological classification does not provide much information about the responsible cause of disease and misdiagnoses may expose patients to immunosuppressive therapy side effects, mistaken genetic counseling, and misguided kidney transplant programs.

## Introduction

Focal segmental glomerulosclerosis (FSGS) represents one of the major causes of nephrotic syndromes (NS) and kidney failure (KF) in the USA, accounting for about 20% of NS cases in children and 40% in adults [1].

Based on data from an international survey, FSGS is predominant in North America, accounting for 19.1% of primary glomerular diseases, while it is less common in Europe, reaching about 15% among a study of 60,300 biopsy reports, resulting less common than IgA nephropathy (22.1%), as expected [2]. The increased incidence in the USA may be associated with the *APOL1* risk genotype in sub-Saharan population, which is underrepresented in other countries. In a Spanish study on 9378 patients affected by NS, FSGS has been reported in 12% of cases, while membranous nephropathy (MN) is the major cause of nephrotic proteinuria in adults, with a prevalence of about 25% [3].

Consistently, in another study from China including 851 patients subjected to kidney biopsy, MN was the most frequent cause of NS (28.8%), followed by other glomerulonephritis, with FSGS affecting 5% of the patients included in the cohort [4]. FSGS is a rare disease. However, in the last 20 years, a worldwide increased prevalence of FSGS has been estimated, probably related to lifestyle and dietary habits for the secondary forms [5]. FSGS is characterized by glomerular injury, usually involving a minority of glomeruli with a segmental solidification of the tuft, deposition of extracellular matrix, and glomerular hyalinosis; moreover, light microscopy typically reveals abundant resorption of lipid droplets in the proximal tubule cells, due to heavy proteinuria. Juxtamedullary glomeruli are the more vulnerable to develop FSGS, rather than the most superficial ones, due to the higher blood flow rates and higher glomerular capillary shear-stress pressure [6]. With disease progression, the lesion may sequentially involve a higher number of glomeruli, with a more diffuse, and global, sclerosis. Overall, the definition of glomerular sclerosis and its characterization has changed considerably. In 1925, a German pathologist, Theodor Fahr, first described the histopathological features of focal segmental hyalinization in a case of “progressive lipoid nephrosis with degeneration” [7], showing association with minimal change disease (MCD), both defined as podocytopathies [8–11]. While the definition of MCD did not change much, over the years, in the mid-1980s, other patterns of glomerular damage have become part of the FSGS spectrum [12]. Even though the term “FSGS” continues to be used as an expression of a diagnosis, it is clear that it may relate to many different conditions, primary and secondary forms, and potentially hiding unexpected genotypes, as it has recently emerged [13–15].

FSGS usually manifests with NS, and patients can be classified as steroid-sensitive NS (SSNS) and as steroid-resistant NS (SRNS) when there is a lack of response to standard treatment with steroids and progressive kidney damage. Thus, it is frequent to consider FSGS and SRNS as synonymous [16, 17]. Monogenic forms of FSGS are more common in children with FSGS/SRNS, with a reported prevalence of about 25% [18–20].

Monogenic FSGS in adults is difficult to estimate because genetic testing does not represent a routine test, but is limited to selected cases with positive family history, relapse or resistance to immunosuppressive therapy, early onset of disease, or the association to extrarenal manifestations, assuming the presence of syndromic conditions. However, genetic defects leading to FSGS also happen in sporadic cases [21].

FSGS is a glomerulonephritis with a not completely clear pattern of injury and it has required special efforts for the understanding of its molecular biology and for the identification of primary and secondary causes. FSGS physiopathology has been progressively investigated, with the aim to increase scientific knowledge and to better define the critical role of the term “glomerulosclerosis” in a renal pathology scenario. The heterogeneity of FSGS and the laborious process to define the range of conditions into which the term FSGS falls have increased the complexity of defining the final and precise diagnosis, making management and treatment options more demanding. Indeed, FSGS does not represent just the description of a single disease, but rather may appear during very different mechanisms of damage. Thus, in case of kidney biopsy suggestive of a FSGS pattern, a complex explorative framework should be applied and it should definitely include genetic testing, to hopefully identify the correct cause and to apply the proper personalized treatment.

The histological definition of FSGS includes a very large disease spectrum and a morphological description is used to identify primary (idiopathic and immunological), secondary, and genetic disorders [22].

Genetic studies played a central role in the identification of genetic variants encoding proteins essential for podocyte structure and function (slit diaphragm components, actin cytoskeleton components, proteins essential for coenzyme Q10 biosynthesis, nuclear proteins, and transcription factors) that can be responsible for NS [23].

Genetic testing has progressively increased the power of discovery for hereditary forms, with more than 60 genes considered monogenic causes of FSGS/NS (Table 1). So, currently, a correct integrated approach should consider clinical data, medical history, family history, renal pathology when available, and a genomic evaluation [29]. This new path of analysis may be tough for clinicians, requiring a deep knowledge of the disease and a larger availability of diagnostic and therapeutic tools, making the genotype–phenotype correlation progressively more challenging.

**Table 1:** Monogenic causes of FSGS/NS

Gene	OMIM code	Protein	MOI	Locus	MIM number Phenotype	Phenotypes	Function	Ref.	Non-syndromic/Syndromic
CD2AP	604241	CD2-associated protein	AD/AR	6p12.3	607832	(FSGS3)	Slit diaphragm and adaptor proteins	Kim, JM [24]	Non-syndromic
NPHS1	602716	Nephrin	AR	19q13.1	256300	(NPHS1)	Slit diaphragm and adaptor proteins	Kestila, M [25]	Non-syndromic
NPHS2	604766	Podocin	AR	1q25.2	600995	(NPHS2)	Slit diaphragm and adaptor proteins	Boute, N [26]	Non-syndromic
PLCE1	608414	Phospholipase C, ε1	AR	10q23	610725	(NPHS3)	Slit diaphragm and adaptor proteins	Hinkes, B [27]	Syndromic/Non-syndromic
ACTN4	604638	α-actinin-4	AD	19q13	603278	Late onset SRNS (FSGS1)	Cytoskeleton components	Kaplan, JM [28]	Non-syndromic
ANLN	616027	Anillin	AD	7p14.2	616032	(FSGS8)	Cytoskeleton components	Lipska-Ziętkiewicz, S [29]	Non-syndromic
ARHGAP24	610586	Arhgap24 (RhoGAP)	AD	4q22.1	N.D.	Adolescent-onset FSGS	Cytoskeleton components	Lipska-Ziętkiewicz, S [29]	Non-syndromic
ARHGDIA	601925	Rho GDP-dissociation inhibitor (GDI) a1	AR	17q25.3	615244	Nephrotic syndrome type 8	Cytoskeleton components	Gee, HY [30]	Syndromic
AVIL	613397	Advillin	AR	12q14.1	618594	Nephrotic syndrome, type 21	Cytoskeleton components	Lipska-Ziętkiewicz, S [29]	Syndromic
INF2	610982	Inverted formin-2	AD	14q32.33	613237-614455	(FSGS5), Charcot-Marie-Tooth disease with glomerulopathy	Cytoskeleton components	Brown, EJ [31]	Syndromic/Non-syndromic
KANK4	614612	KN motif and ankyrin repeat domain-containing protein 4	AR	1p31.3	N.D.	Steroid-resistant nephrotic syndrome	Cytoskeleton components	Gee, HY [32]	Non-syndromic
MYH9	160775	Myosin, heavy chain 9	AD	22q12.3	603622-155100	Deafness, autosomal dominant 17, Macrothrombocytopenia and granulocyte inclusions with or without nephritis or sensorineural hearing loss	Cytoskeleton components	Lipska-Ziętkiewicz, S [29]	Syndromic
MYO1E	601479	Myosin IE	AR	15q22.2	614131	Childhood-onset SRNS (FSGS6)	Cytoskeleton components	Mele, C [33]	Non-syndromic
WDR73	616144	WD repeat domain 73	AR	15q25.2	251300	Galloway-Mowat syndrome 1	Cytoskeleton components	Lipska-Ziętkiewicz, S [29]	Syndromic
DLC1	604258	Rho gtpase-activating protein 7	N.D.	8p22		Colorectal cancer, somatic; Nephrotic syndrome	Cytoskeleton components	Lipska-Ziętkiewicz, S [29]	Non-syndromic
CRB2	609720	Crumbs homolog 2	AR	9q33.3	616220-219730	(FSGS9), Ventriculomegaly with cystic kidney disease	Apical membrane proteins	Lipska-Ziętkiewicz, S [29]	Syndromic/Non-syndromic
TRPC6	603652	Transient receptor potential channel 6	AD	11q22.1	603965	(FSGS2)	Apical membrane proteins	Winn, MP [34]	Non-syndromic
EMP2	602334	Epithelial membrane protein 2	AD	16p13.2	615861	(NPHS10)	Apical membrane proteins	Gee, HY [35]	Non-syndromic
MXRA5	300938	Matrix-remodeling-associated protein 5	XLR	Xp22.33	N.D.	Steroid-resistant nephrotic syndrome	Apical membrane proteins	Warejko, K [19]	Non-syndromic
PODXL	602632	Podocalyxin	AD	7q32.3	N.D.	Steroid-Resistant Nephrotic Syndrome	Apical membrane proteins	Barua, M [36]	Non-syndromic
CD151	602243	Tetraspanin	AR	11p15.5	609057-179620	Epidermolysis bullosa simplex 7 with nephropathy and deafness, [Blood group, Raph]	GBM and basal membrane proteins and related components	Lipska-Ziętkiewicz, S [29]	Syndromic
EXT1	608177	Glycosyltransferase	AR	8q24.11	133700	Exostoses multiple type 1	GBM and basal membrane proteins and related components	Tae-Sun Ha [37]	Non-syndromic
ITGA3	605025	Integrin-β3	AR	17q21.33	614748	Epidermolysis bullosa junctional 7 with interstitial lung disease and nephrotic syndrome	GBM and basal membrane proteins and related components	Has, C [38]	Syndromic
ITGB4	147557	Integrin-β4	AR	17q25.1	619816-226730	Epidermolysis bullosa junctional 5A intermediate, Epidermolysis bullosa junctional 5B with pyloric atresia	GBM and basal membrane proteins and related components	Lipska-Ziętkiewicz, S [29]	Syndromic
SCARB2	602257	Scavenger receptor class B, member 2 (LIMP II)	AR	4q21.1	254900	Epilepsy progressive myoclonic 4 with or without renal failure	Lysosomal proteins	Lipska-Ziętkiewicz, S [29]	Syndromic
ADCK4 (COQ8B)	615567	AarF domain containing kinase 4	AR	19q13.1	615573	Nephrotic syndrome type 9	Mitochondrial proteins	Ashraf, SA [39]	Non-syndromic
COQ2	609825	4-hydroxybenzoate polyprenyltransferase	AR/AD	4q21.23	607426-146500	Coenzyme Q10 deficiency primary 1, {Multiple system atrophy, susceptibility to}	Mitochondrial proteins	Diomedi-Camassei, F [40]	Syndromic
COQ6	614647	Ubiquinone biosynthesis monooxygenase COQ6	AR	14q24.3	614650	Coenzyme Q10 deficiency primary 6	Mitochondrial proteins	Heeringa, SF [41]	Syndromic
MTTL1	590050	Mitochondrially encoded tRNA leucine 1 (UUA/G)	Maternal	mtDNA	N.D.	Mitochondrial diabetes, deafness with FSGS , MELAS syndrome	Mitochondrial proteins	Tae-Sun Ha [37]	Syndromic
PDSS2	610564	Decaprenyl-diphosphate synthase subunit 2	AR	6q21	614652	Coenzyme Q10 deficiency primary 3	Mitochondrial proteins	Lopez, LC [42]	Syndromic
DDX53	301079	Probable ATP-dependent RNA helicase DDX53	XLR	Xp22.11	N.D.	Steroid-resistant nephrotic syndrome	Nuclear proteins	Warejko, K [19]	Non-syndromic
GATA3	131320	GATA binding protein 3	AD	10p14	146255	HDR syndrome (hypoparathyroidism, sensorineural deafness, renal abnormalities)	Nuclear proteins	Tae-Sun Ha [37]	Syndromic
LAGE3	300060	EKC/KEOPS complex subunit LAGE3	XLR	Xq28	301006	Galloway-Mowat syndrome 2 X-linked	Nuclear proteins	Lipska-Ziętkiewicz, S [29]	Syndromic/Non-syndromic
MAFB	608968	A transcription factor	AD	20q12	617041-166300	Duane retraction syndrome 3, Multicentric carpotarsal osteolysis syndrome	Nuclear proteins	Tae-Sun Ha [37]	Syndromic
NUP107	607617	Nucleoporin 107kD	AR	12q15	618078-618348-616730	Ovarian dysgenesis 6, Galloway-Mowat syndrome 7, Nephrotic syndrome type 11	Nuclear proteins	Lipska-Ziętkiewicz, S [29]	Syndromic
NUP205	614352	Nuclear pore complex protein Nup205	AR	7q33	616893	Nephrotic syndrome type 13	Nuclear proteins	Braun, DA [43]	Syndromic
NUP85	170285	Nuclear pore complex protein Nup85	AR	17q25.1	618176	Nephrotic syndrome type 17	Nuclear proteins	Lipska-Ziętkiewicz, S [29]	Syndromic
NUP93	614351	Nucleoporin 93kD	AR	16q13	616892	Nephrotic syndrome, type 12	Nuclear proteins	Braun, DA [43]	Non-syndromic
NXF5	300319	Nuclear RNA export factor 5	XR	Xq21	N.D.	SRNS/FSGS cardiac conduction disorder	Nuclear proteins	Lipska-Ziętkiewicz, S [29]	Syndromic
OSGEP	610107	tRNA N6-adenosine threonylcarbamoyltransferase	AR	14q11.2	617729	Galloway-Mowat syndrome 3	Nuclear proteins	Lipska-Ziętkiewicz, S [29]	Syndromic
SMARCAL1	606622	HepA-related protein	AR	2q35	242900	Schimke immuno-osseous dysplasia	Nuclear proteins	Boerkoel, CF [44]	Syndromic
TPRKB	608680	EKC/KEOPS complex subunit TPRKB	AR	2p13.1	617731	Galloway-Mowat syndrome 5	Nuclear proteins	Warejko, K [19], Lipska-Ziętkiewicz, S [29]	Syndromic
WT1	607102	Wilms’ tumor protein	AD/AR	11p13	194080-136680-608978-156240-256370-194070	Denys-Drash syndrome, Frasier syndrome, Meacham syndrome, Mesothelioma somatic, Nephrotic syndrome type 4, Wilms tumor type 1	Nuclear proteins	Tae-Sun Ha [37]	Syndromic/Non-syndromic
XPO5	607845	Exportin-5	AR	6p21.1	N.D.	Steroid-Resistant Nephrotic Syndrome	Nuclear proteins	Braun, DA [43]	Non-syndromic
ALG1	605907	β1,4 mannosyltransferase	AR	16p13.3	608540	Congenital disorder of glycosylation type Ik	Other intracellular proteins	Lipska-Ziętkiewicz, S [29]	Syndromic
ANKFY1	607927	Rabankyrin-5	N.D.	17p13.2	N.D.	Genetic steroid-resistant nephrotic syndrome	Other intracellular proteins	Lipska-Ziętkiewicz, S [29]	Non-syndromic
APOL1	603743	Apolipoprotein L1	AR	22q12.3	612551	FSGS in African-Americans (FSGS4)	Other intracellular proteins	Lipska-Ziętkiewicz, S [29]	Non-syndromic
CDK20	610076	Cyclin-dependent kinase 20	AR	9q22.1	N.D.	Steroid resistant nephrotic syndrome	Other intracellular proteins	Warejko, K [19];	Non-syndromic
COG1	606973	Conserved oligomeric Golgi complex subunit 1	AR	17q25.1	611209	Congenital disorder of glycosylation, type Iig	Other intracellular proteins	Warejko, K [19]	Non-syndromic
DGKE	601440	Diacylglycerol kinase-ε	AR	17q22	615008	Nephrotic syndrome type 7, {Hemolytic uremic syndrome, atypical, susceptibility to, 7}	Other intracellular proteins	Lipska-Ziętkiewicz, S [29]	Syndromic
DHTKD1	614984	2-oxoadipate dehydrogenase complex component E1	AD/AR	10p14	615025-204750	Charcot-Marie-Tooth disease axonal type 2Q, Alpha-aminoadipic and alpha-ketoadipic aciduria	Other intracellular proteins	Warejko, K [19]	Syndromic
E2F3	600427	Transcription factor E2F3	AD	6p22.3	N.D.	ID (whole-gene deletion)	Other intracellular proteins	Lipska-Ziętkiewicz, S [29]	Syndromic
KANK2	614610	KN motif and ankyrin repeat domain-containing protein 2	AR	19p13.2	617783-616099	Nephrotic syndrome type 16, Palmoplantar keratoderma and woolly hair	Other intracellular proteins	Gee, HY [32]	Non-syndromic
MAGI2	606382	Membrane-associated guanylate kinase, ww and pdz domains-containing, 2	AR	7q21.11	617609	Nephrotic syndrome type 15	Other intracellular proteins	Lipska-Ziętkiewicz, S [29]	Syndromic
PAX2	167409	Paired box protein Pax-2	AD	10q24.31	616002-120330	Glomerulosclerosis focal segmental 7, Papillorenal syndrome	Other intracellular proteins	Becherucci, F [45]	Syndromic
PMM2	601785	Phosphomannomutase 2	AR	16p13.2	212065	Congenital disorder of glycosylation type Ia	Other intracellular proteins	Lipska-Ziętkiewicz, S [29]	Syndromic
PTPRO	600579	Tyrosine phosphatase receptor-type O (GLEPP1)	AR	12p12.3	614196	(NPHS6)	Other intracellular proteins	Lipska-Ziętkiewicz, S [29]	Non-syndromic
SGPL1	603729	Sphingosine-1-phosphate lyase 1	AR	10q22.1	617575	Nephrotic syndrome type 14	Other intracellular proteins	Lipska-Ziętkiewicz, S [29]	Syndromic
SLC35F1	620349	Solute carrier family 35 member F1	AR	6q22.2	N.D.	Steroid resistant nephrotic syndrome	Other intracellular proteins	Warejko, K [19]	Non-syndromic
TTC21B	612014	IFT139 (a component of intraflagellar transport- A)	AD/AR	2q24.3	613820-613819	Nephronophthisis 12, Short-rib thoracic dysplasia 4 with or without polydactyly	Other intracellular proteins	Lipska-Ziętkiewicz, S [29]	Non-syndromic
GAPVD1	611714	GTPase-activating protein and vps9 domains 1	N.D.	9q33.3	N.D.	Steroid-resistant nephrotic syndrome	Other intracellular proteins	Hermle, T [46]	Non-syndromic

*MOI* Mode of Inheritance; *AD* Autosomal Dominant; *AR* Autosomal Recessive; *Ref*. Reference

Earlier genetic studies of FSGS used positional cloning mapping [25, 27] applied to large families with multiple affected family members, and targeted single gene sequencing technology to detect causal mutations in already established NS genes.

Those approaches have been useful to identify rare mutations in single genes highly expressed in podocytes and among the glomerular filtration barrier [47–49]. However, it traditionally required time-consuming and non-cost-effective Sanger sequencing validations, representing a sensitive technique but more problematic when applied to heterogeneous disorders caused by multiple genes [50, 51].

Thanks to the advent of recent applications of high-throughput technologies, using massive parallel sequencing, the strategies used to analyze the genomic background of patients affected by complex diseases, such as FSGS/SRNS, have completely changed [52, 53]. Next-generation sequencing (NGS) technologies include the analysis of (a) targeted panels of genes of interest (eventually selected based on hypothesis-driven approach), (b) the coding exonic regions (whole exome sequencing (WES)), or (c) the entire coding and non-coding genomic background (whole genome sequencing (WGS)). Those methods have dramatically increased the power of capturing disease-causing mutations [51], with a broader spectrum of diagnostic yield, not only including pathogenic variants in well-known NS genes, but also leading to discovery of unexpected genotypes.

Consequent to the introduction of these new tools, clinicians may investigate through larger lenses the genomic background in FSGS/SRNS patients, with promising results and new scenarios, performing a more comprehensive diagnosis. Furthermore, massive sequencing analysis has also raised the possibility of dealing with the discovery of unpredictable pathogenic mutations, leading to consideration of new genotype–phenotype correlations and the chance of thinking outside the classical pathways of disease. Unfortunately, not all centers have the availability of expensive NGS applications, thus often, genetic testing remains only applied to selected cases.

While the term “phenocopy” was introduced in medicine about 75 years ago, recently it has been subjected to conceptual expansion in human diseases [54]. A progressively increasing number of genes known to be associated with different disorders, acting as phenocopies, have emerged from sequencing data from FSGS/SRNS cohorts (Table 2).

**Table 2:** Phenocopies of FSGS/SRNS

Gene	OMIM code	Protein	MOI	Locus	MIM number Phenotype	Phenotypes	Function	Reference
AGXT	604285	Alanine--glyoxylate aminotransferase	AR	2q37.3	259900	Hyperoxaluria primary type 1	Lysosomal proteins	Becherucci, F [45]
CFH	134370	Complement factor H	AD/AR	1q31.3	126700-609814-235400-610698	Basal laminar drusen, Complement factor H deficiency, {Hemolytic uremic syndrome, atypical, susceptibility to, 1}, {Macular degeneration, age-related, 4}	Other intracellular proteins	Lipska-Ziętkiewicz, S [29]
CLCN5	300008	H(+)/Cl(-) exchange transporter 5	XLD	Xp11.23	308990-310468-300554-300009	Proteinuria, low molecular weight with hypercalciuric nephrocalcinosis, Nephrolithiasis type I, Hypophosphatemic rickets, Dent disease I	Other intracellular proteins	Becherucci, F [45]
COL4A3	120070	Collagen alpha-3(IV) chain	AR/AD	2q36.3	203780-104200-620320	Alport syndrome 2 autosomal recessive, Alport syndrome 3 autosomal dominant, Hematuria, benign familial 2	GBM and basal membrane proteins and related components	Becherucci, F [45]
COL4A4	120131	Collagen alpha-4(IV) chain	AR/AD	2q36.3	203780-141200	Alport syndrome 2 autosomal recessive, Hematuria familial benign 1	GBM and basal membrane proteins and related components	Mochizuki, T. [55]
COL4A5	303630	Collagen alpha-5(IV) chain	XLD	Xq22.3	301050	Alport syndrome 1 XLD	GBM and basal membrane proteins and related components	Becherucci, F [45]
CTNS	606272	Cystinosin	AR	17p13.2	219750-219800-219900-219800	Cystinosis ocular nonnephropathic, Cystinosis nephropathic, Cystinosis late, onset juvenile or adolescent nephropathic, Cystinosis atypical nephropathic	Lysosomal proteins	Becherucci, F [45]
CUBN	602997	Cubilin	AR	10p13	261100-618884	Imerslund-Grasbeck syndrome 1, [Proteinuria, chronic benign]	Other intracellular proteins	Lipska-Ziętkiewicz, S [29]
FAT1	600976	Fat atypical cadherin 1	AR	4q35.2	N.D.	FAT1-related glomerulotubular nephropathy	Apical membrane proteins	Becherucci, F [45]
FAT4	612411	Protocadherin Fat 4	AR	4q28.1	615546-616006	Van Maldergem syndrome 2, Hennekam lymphangiectasia-lymphedema syndrome 2	Apical membrane proteins	Becherucci, F [45]
FN1	135600	Fibronectin	AD	2q35	601894-184255	Glomerulopathy with fibronectin deposits 2, Spondylometaphyseal dysplasia corner fracture type	Cytoskeleton components	Castelletti, F [56]
GLA	300644	Alpha-galactosidase A	XLD	Xq22.1	301500	Fabry disease (cardiac variant)	Lysosomal proteins	Lipska-Ziętkiewicz, S [29]
KANK1	607704	KN motif and ankyrin repeat domain-containing protein 1	AR	9p24.3	612900	Cerebral palsy spastic quadriplegic 2	Cytoskeleton components	Becherucci, F [45]
LAMB2	150325	Laminin subunit beta-2	AR	3p21.31	614199-609049	Nephrotic syndrome type 5 with or without ocular abnormalities, Pierson syndrome	GBM and basal membrane proteins and related components	Becherucci, F [45]
LCAT	606967	Phosphatidylcholine-sterol acyltransferase	AR	16q22.1	136120-245900	Fish-eye disease, Norum disease	Other intracellular proteins	Lipska-Ziętkiewicz, S [29]
LMNA	150330	Prelamin-A/C	AD	1q22	151660	Lipodystrophy familial partial type 2	Nuclear proteins	Lipska-Ziętkiewicz, S [29]
LMX1B	602575	LIM homeobox transcription factor 1-beta	AD	9q33.3	161200-256020	Nail–patella syndrome, Focal segmental glomerulosclerosis 10	Nuclear proteins	Becherucci, F [45]
LRP2	600073	Low-density lipoprotein receptor-related protein 2	AR	2q31.1	222448	Donnai-Barrow syndrome	Lysosomal proteins	Kantarci, S [57]
MEFV	608107	Pyrin	AD/AR	16p13.3	608068-249100-134610	Neutrophilic dermatosis acute febrile, Familial Mediterranean fever	Other intracellular proteins	Warejko, K [19]
MMACHC	609831	Cyanocobalamin reductase / alkylcobalamin dealkylase	AR	1p34.1	277400	Methylmalonic aciduria and homocystinuria cblC type	Other intracellular proteins	Lipska-Ziętkiewicz, S [29]
NEU1	608272	Oxytocin-neurophysin 1	AR	6p21.33	256550	Sialidosis type I-II	Other intracellular proteins	Lipska-Ziętkiewicz, S [29]
NPHP4	607215	Nephrocystin-4	AR	1p36.31	606966-606996	Nephronophthisis 4, Senior-Loken syndrome 4	Apical membrane proteins	Lipska-Ziętkiewicz, S [29]
OCRL	300535	Inositol polyphosphate 5-phosphatase OCRL	XLR	Xq26.1	309000-300555	Lowe syndrome, Dent disease 2	Cytoskeleton components	Attree, O [58]
WDR19	608151	WD repeat-containing protein 19	AR	4p14	614377	Nephronophthisis 13	Cytoskeleton components	Becherucci, F [45]
ZMPSTE24	606480	CAAX prenyl protease 1 homolog	AR	1p34.2	608612-275210	Mandibuloacral dysplasia with type B lipodystrophy, Restrictive dermopathy 1	Nuclear proteins	Lipska-Ziętkiewicz, S [29]

*MOI* Mode of Inheritance; *AD* Autosomal Dominant; *AR* Autosomal Recessive; *Ref*. Reference

So, if it is true that FSGS heterogeneity is well established, and if the main morphologic features are the focal fibrosis and glomerular scars, what lies behind this? How should we consider a pattern of glomerulosclerosis in this new heterogeneous fashion? For many years, the main interest in the genetics of FSGS/SRNS has been podocyte-centric, maintaining the interest of clinicians and geneticists among podocyte and glomerular filtration barrier components.

Progressively, new classes of genes have been described in cohorts of NS/FSGS patients, not directly associated with podocyte function and structure, but associated with different mechanisms of kidney damage, that may not primarily cause NS, but mimic FSGS and SRNS.

In this paper, we want to emphasize how new genetic insights have demonstrated that FSGS is a complex disease, characterized by “many masks,” and requiring a new “open vision” in its etiological investigation and clinical management. A new critical and integrated approach is needed in order to obtain the most accurate genotype–phenotype correlation.

## Classification evolution: background of the FSGS issue

FSGS comprises a group of clinical-pathologic conditions clinically characterized by heavy proteinuria, hyperlipidemia, edema, and hypoalbuminemia with histological features of obliteration of glomerular capillaries by extracellular matrix with segmental distribution. However, FSGS lesions are heterogeneous. In general, primary FSGS refers to the idiopathic form with severe proteinuria, without a specific or labeled cause and for which circulating immunological triggers have been considered, even if not yet clearly identified. Secondary forms include those FSGS cases related to specific and recognizable causes, such as viral infections, drugs/toxin exposure, maladaptive nephron response, and genetic mutations.

In 2004, a working group of international renal pathologists convened at Columbia University to define and formulate the features of histological patterns of FSGS to create a histopathological classification of the disease. Columbia classification was born with the aim to define morphological criteria for the different pathological features of FSGS, for primary as well as secondary forms. It became a guide to use standardized pathological nomenclature. Five mutually exclusive morphologic variants were described, differentiating FSGS in (a) tip lesion, (b) cellular, (c) perihilar, (d) collapsing, and (e) not otherwise specified (NOS) variants (Table 3), referring to both primary and secondary forms of FSGS [59–61].

**Table 3:** Columbia classification

Variant	Description
FSGS (NOS - Not Otherwise Specified)	When identified at least 1 glomerulus with segmental increase in matrix obliterating the capillary lumina, without podocyte hyperplasia, and often areas of adhesion to Bowman’s capsula. Exclude perihilar, cellular, tip, and collapsing variants
Perihilar variant	When identified at least 1 glomerulus with perihilar hyalinosis, with or without sclerosis >50% of glomeruli with segmental lesions, with perihilar distribution of sclerosis and/or hyalinosis. Often sign of adhesion and glomerulomegaly. Exclude cellular, tip, and collapsing variants
Cellular variant	When identified at least 1 glomerulus with endocapillary hypercellularity occluding lumina with segmental distribution, with or without foam cells and karyorrhexis. Lesion may be located anywhere among the gloms structure. Exclude tip and collapsing variants.
Tip variant	When identified at least 1 segmental lesion involving the tip domain (outer 25% of tuft next to origin of proximal tubule) or cellular (in <50% of tuft). The tubular pole must be identified in the defining lesion. An adhesion or confluence of podocytes with parietal or tubular cells at the tubular lumen or neck, should be identified. Lesions can be sclerosing (in <25% of tuft). Exclude collapsing and any perihilar sclerosis.
Collapsing variant	When identified at least 1 glomerulus with segmental or global collapse and overlying podocyte hypertrophy and hyperplasia, often with podocyte droplets/vacuoles. Distribution may be segmental or global.

Modified from D D'Agati, V., Fogo, A. B., Bruijn, J. A., & Jennette, J. C. (2004). Pathologic classification of focal segmental glomerulosclerosis: a working proposal. American journal of kidney diseases, 43(2), 368-382

This morphological description led to new considerations of this disease, no longer limited to provide a pure description of kidney biopsy, but rather placed in a broader setting of new clinical-pathological scenario. Thus, the first FSGS classification was an excellent starting point to develop new future studies with the aim of understanding the molecular mechanisms differentiating FSGS variants, their different outcome and clinical progression, starting from morphological heterogeneity and trying to reduce the ambiguous use of the term “FSGS”.

While the Columbia classification aimed towards a morphological description of proliferative and sclerosing histopathological patterns with no specific correlation to pathogenesis and no contribution to treatment options, to implement the pure descriptive features with etiology and pathogenesis correlations, in 2007, the term “taxonomy” was first introduced to define an integrated and multiple-level analysis in the spectrum of heterogeneous FSGS disease [62]. The aim of the taxonomy of podocytopathies was to provide a categorization of patterns of podocyte injuries describing FSGS as a well-characterized glomerular disease due to the grade of sustained podocyte rearrangement, detachment, and apoptosis. In this new classification, the podocyte, being the main site of damage, takes the center stage, becoming the major protagonist to differentiate the kind of damage. The new approach to classification of podocytopathies distinguished four different glomerular pathways of injury, considering podocyte number modification and integrating morphological features with etiology, including idiopathic, genetic, and reactive forms. This new classification included (a) *minimal change nephropathy* (MCN) characterized by podocyte injury without modification of podocyte number,(b) *focal segmental glomerulosclerosis* (FSGS) with loss of podocytes, cell death, and insufficient repair activity; (c) *diffuse mesangial sclerosis* (DMS) characterized by mesangial expansion with mild proliferation, podocyte hypertrophy and hyperplasia, and lower degree of cell differentiation; and (d) *collapsing glomerulopathy* (CG) characterized by collapse of the glomerular tuft in at least one glomerulus with hyperplasia and hypertrophy of de-differentiated podocytes, leading to pseudo-crescent formation [62] (Table 4).

**Table 4:** Taxonomy of Podocytopathies

Variant	Description
Minimal Change Nephropathy (MCN)	No changes are present on light microscopy. Normal histology. No change in podocyte number.
Focal Segmental Glomerulosclerosis (FSGS)	Segmental solidification of the tuft with accumulation of extracellular matrix. Synechiae between the tuft and Bowman’s capsule. podocytes are lost in the areas of sclerosis. Activation of apoptotic pathway with podocytopenia due to cell death.
Diffuse Mesangial Sclerosis (DMS)	Mesangial expansion resulting from accumulated extracellular matrix, accompanied by mild proliferation of hypertrophic podocytes, due to development arrest.
Collapsing Glomerulopathy (CG)	Wrinkling and folding of the glomerular basement membranes with collapse and proliferation of de-diffentiated podocytes, leading to pseudo-crescents formation. Numerous protein reabsorption droplets are present in the podocytes.

Modified from Barisoni, L., Schnaper, H. W., & Kopp, J. B. (2007). A proposed taxonomy for the podocytopathies: a reassessment of the primary nephrotic diseases. Clinical Journal of the American Society of Nephrology, 2(3), 529-542

Even if the description of FSGS has gained more deep knowledge and a better diagnostic approach, over the years, its definition and classification are still subjected to evolution and updates.

The recent KDIGO guidelines, published in 2021, have proposed a new, more recent classification for FSGS that distinguishes four groups, based on light microscopy lesions, in order to improve clinical and treatment management. The aim of the new classification was to integrate a more comprehensive pathophysiology meaning and treatment options. The updated nomenclature includes (a) *primary FSGS* with extensive foot process effacement and sudden NS, usually linked to permeability factors still investigated and not yet well established; (b) *genetic forms* for all the familial, sporadic, and syndromic conditions due to pathogenic mutation in autosomal dominant or recessive or X-linked genes, known to be associated with FSGS and NS; (c) *secondary forms*, referring to the viral and toxic-induced FSGS cases, and including also the maladaptive conditions caused by normal or reduced nephron mass, often associated with segmental foot process effacement and milder proteinuria; and (d) *FSGS of undetermined causes (FSGS-UC)* in which all the other cases of unknown origin are included [63] (Table 5).

**Table 5:** KDIGO FSGS classification

Primary FSGS	Characterized by idiopathic nephrotic syndrome with diffuse foot process effacement without any identified cause explaining the disease.
Genetic FSGS	All those FSGS in which a pathogenic mutation lead to the disease. It may be Familial, Sporadic, Syndromic. About 60 genes have been identified in association to FSGS, so far.
Secondary FSGS	Usually characterized by segmental foot process effacement, milder proteinuria without nephrotic syndrome, caused by one of the following: Viral infections, Drugs/Toxin Exposition, Maladaptive condition due to normal or reduced Nephron Mass.
FSGS of Undetermined cause (FSGS-UC)	When any of the other causes are identified. It is characterized by segmental foot process effacement and milder proteinuria without nephrotic syndrome.

Modified from: Kidney Disease: Improving Global Outcomes (KDIGO) Glomerular Diseases Work Group. KDIGO 2021 Clinical Practice Guideline for the Management of Glomerular Diseases. Kidney Int. 2021 Oct;100(4S):S1-S276. 10.1016/j.kint.2021.05.021. PMID: 34556256

Thus, in the last 20 years, the description and the classification of FSGS have deeply evolved, from the pure morphological descriptions to an integrated classification based on podocyte fate and disease progression, until the most recent etiological classification with potential treatment options.

Nephrologists, pathologists, and scientists have focused on the role of the podocyte as the cell primarily involved in the regulation of glomerular filtration homeostasis. Therefore, a lot of advancements have been made in understanding the biology of podocyte cells and the role of genetic modifications altering the glomerular cell balance.

So far, approximately 60 genes have been described in association with podocytopathies and NS (Table 1), representing the main clinical sign of primary FSGS and also the second most common cause of chronic kidney disease (CKD) in children and young adults less than 25 years old [64].

During the first years of the twenty-first century, a common idea was that podocyte damage was involved in different forms of human and experimental glomerular disease, such as MCD, FSGS, CG, and membranous and diabetic nephropathies, all of which diseases are related to clinical manifestation of NS. Thanks to the growing interest of the research community in understanding the pathogenesis of the different forms of glomerulonephritis, today glomerular diseases have been better characterized, providing new knowledge in terms of the molecular, immunological, and genetic mechanisms.

Epithelial visceral cells directly regulate the glomerular filtration rate. Most of the diseases caused by abnormal glomerular cell function are characterized by podocyte injuries and/or dysfunction [9, 65–68]. Podocytopathies are then defined as a group of diseases, including FSGS and MCD, characterized by structural and functional podocyte impairment causing NS as an expression of glomerular filtration barrier damage. However, this classification also has been subjected to a progressive evolution, since new insights about FSGS have been achieved.

Since nephrin (NPHS1) was first described as causing early-onset NS, new observations and updated understanding have occurred. Thanks to massive parallel DNA sequencing technologies, FSGS is no longer an area of localized sclerosis associated with podocyte defects. While initially the major genes causing monogenic forms of NS/FSGS described were those associated with defects in podocytes, slit diaphragm, glomerular basement membrane (GBM), or altering actin remodeling resulting in podocyte dysfunction [37, 69, 70], in recent years, also, genes encoding proteins working far from the glomerulus have been added to the list of genes that can potentially cause FSGS. In a study from 2018, including 300 patients affected by SRNS and subjected to WES, the authors found phenocopies in 5% of the cohort. This has been the first report applying the concept of *phenocopy* to SRNS, explaining the possibility that FSGS may be associated with mutation in genes that do not purely affect podocytes, even if leading to proteinuria [19].

## FSGS glomerular scars

In a single kidney biopsy, the features of FSGS may be wide. Kidney sampling may be tricky and sometimes it is possible that sclerotic glomeruli may be unsampled, resulting in specimens with normal glomeruli at light microscopy evaluation, but extensive foot process effacement at electron microscopy. This event should always predict the possibility of a sclerotic pattern in the glomeruli not collected. Proteinuria is one of the most common clinical findings in FSGS. Renal pathology in FSGS patients, as well as other proteinuric glomerular diseases, has demonstrated the importance of podocyte structure and glomerular barrier in the homeostasis of the urine filtration mechanism. The glomerular filtration barrier is composed of podocytes, GBM, and endothelial cells. A crosstalk between podocytes and endothelial cells exists, through the production of vascular endothelial grow factor (VEGF) by podocytes [71].

Interestingly, studies on animal models using the NEP25 chimeric mouse, in which only some podocytes express the toxin receptor human CD25, suggest that podocyte injury can extend from receptor-positive to receptor-negative podocytes, due to a different hypothesis: damaged podocytes may release toxic molecules such as chemokines, TGF-β, endothelin-1 altering podocyte survival, and also reduce the concentration of cell protective factors such as VEGF, due to altered environment after podocyte death. Reduced podocyte survival may also result from loss of podocyte–podocyte interactions or apoptosis signals through altered gap junctions, coming from damaged podocytes. Consequently, podocyte-to-podocyte damage transmission has been hypothesized as one of the mechanisms to explain the progressive extension of glomerulosclerosis, resulting from both direct and indirect triggers [72].

Glomerular scars and extracellular matrix distribution may result in the following three main events: (1) matrix deposition involves the glomerulus directly, as a response to inflammatory injuries caused by systemic inflammatory diseases with necrotizing insults, like vasculitis or lupus nephritis; (2) matrix deposition among the mesangium and GBM, usually where extracellular matrix already exists, with the aim of preserving the glomerular structures, as may happen in diabetic nephropathy and amyloidosis; (3) matrix deposition occurs in capillary loops in the setting of the glomerulus, in conditions like primary FSGS or benign nephroangiosclerosis [73].

The mechanism of glomerular scar formation is still under evaluation, but it has been described as involving cytokines such as TGF-β. TGF-β has been demonstrated to be overexpressed in glomerular disease with podocyte dysfunction [74]. The proteinuria activates molecules responsible for epithelial-to-mesenchymal trans-differentiation in the tubulo-interstitial compartment and the further generation of profibrotic cytokines and inflammatory molecules, to step through damaged podocytes [75, 76].

In addition, damaged tubular cells activate the renin angiotensin system with increased levels of angiotensin II, which acts on mesangial cells by activating them and consequently causing the production of extracellular matrix, through transcription factor “sterol-responsive element-binding protein” (SREBP-1), and finally leading to TGF-β1 upregulation with profibrogenic stimuli [77].

Schiffer et al. evaluated the role of TGF-β and SMAD family proteins in the apoptosis of podocytes and the development of glomerulosclerosis. Using TGF-β transgenic mice and cultured murine podocytes treated with TGF-β, the authors demonstrated that both TGF-β and SMAD7 cause apoptosis of the podocytes but with a different mechanism: while TGF-β activates of mitogen-activated protein (MAP) kinase p38 and classic effector caspase-3, SMAD7 inhibits the NF-κB pathway (nuclear factor kappa-light-chain-enhancer of activated B cells), enhancing the apoptotic activity of TGF-β and therefore the development and progression of glomerulosclerosis [78].

Immunohistochemistry and in situ hybridization analysis in idiopathic FSGS kidney biopsies also demonstrated the involvement of thrombospondin-1 (TSP-1), TGF-β type II receptor (TGF-βIIR) in the increased production of extracellular matrix, through SMAD signaling [79].

The role of TGF-β in the development of glomerular diseases is also evidenced by the finding of elevated TGF-β levels in urine from 42 patients with glomerulonephritis compared to 11 healthy patients, as descripted by Murakami et al. [80].

Although scars are a simple and common histological lesion in various glomerular pathologies, the mechanisms are not entirely clear. There is a complex molecular pathway, both from a biochemical and etiopathogenetic point of view, that can contribute to the development of glomerular sclerosis. With increasing use of precision medicine tools and next-generation sequencing, it has been progressively discovered how glomerular scars are not the expression of a unique exclusive event or just podocyte-related. Thus, FSGS heterogeneity can be considered an opportunity to interpret and to solve new molecular pathways able to influence the clinical manifestations of the disease. FSGS may occlude various etiopathogenetic causes that we need to explore in case of idiopathic, unknown origin, and potentially hereditary cases.

### Genetics of FSGS

Table 1 lists the monogenic causes of FSGS/NS. Over 60 genes have been described as causative of FSGS/SRNS, classified based on the affected glomerular pathway.

Hereditary FSGS should be suspected when it is reported with positive family history, early-onset disease, in case of extrarenal phenotypes, and rapid decline of kidney function or lack of treatment response. When dominant genes are mutated, there is segregation of the disease through generations, while recessive forms usually show absent expression of the disease between generations, with unaffected healthy parents, being heterozygous carriers of the recessive allele or completely healthy in the case of de novo mutations. Clinicians should always investigate the presence of extra-renal manifestations, due to the possibility of syndromic genetic forms of disease, in which NS or a wide range of proteinuria could be associated, such as for example deafness, ocular abnormalities, heart defects, or other nonspecific systemic manifestations, as may happen in complex systemic disorders such as Fabry disease [81]. Incomplete penetrance and variable expression complicate this scenario, with the possibility of having asymptomatic patients while others show a wide spectrum of manifestations, from a mild phenotype with low-grade proteinuria to severe NS and its complications, to progressive CKD and KF, even when having the same genetic mutation. Very little information is available regarding the correlation between genetic FSGS and clinical/histological features. The different classes of FSGS categorize the type and grade of podocyte injuries, going from depletion to apoptosis, to de-differentiation of podocytes, but this morphological classification does not provide any information about the cause of kidney damage, the different pathways leading to disease, recognizable clinical manifestations, or prognostic orientation.

Specific correlations between genetic mutations in FSGS and renal pathology features have not yet been described. Thus, the discrimination between hereditary forms and primary FSGS is pretty difficult. The variable penetrance and expressivity in monogenic FSGS/SRNS explain the difficulty to establish when a patient with FSGS/SRNS would need genetic testing, with challenging individualization of a precise molecular diagnosis, even in the most experienced nephrology clinical setting.

The majority of NS-associated genes are autosomal recessive. *NPHS1* (OMIM 602716) encodes nephrin, an immunoglobulin protein, which represents the hallmark of genetic NS/FSGS, being the first recessive gene discovered to be causative for congenital nephrotic syndrome (CNS) in the Finnish population in 1998. It is the most frequent cause of early-onset NS accounting 40 to 60% of CNS [25, 82], however, mutations in this gene may also occur in sporadic FSGS [83].

Proximal tubular dilatation may be found in kidney biopsy of patients with *NPHS1* mutations [84]. Since nephrin was identified, many genes have been consequently discovered, mapping podocyte and glomerular filtration barriers.

*NPHS2* (OMIM 604766) encodes for podocin, a transmembrane protein located in intracellular podocyte junctions, closely working with NPHS1 and CD2AP OMIM 604241 in the regulation of the slit diaphragm. Interestingly, a variable association has been reported between type of mutations and histological features in *NPHS2*-associated FSGS. While truncating variants in *NPHS2* have been reported with a DMS phenotype, “less” deleterious missense mutations would be more frequently associated with an FSGS phenotype, demonstrating that renal pathology may depend upon the “developmental era” in which a specific gene mutation occurs [85]. Also, the variant p.R229Q is considered an *NPHS2* polymorphism, with a high frequency (about 3%) in non-Finnish Europeans, but becoming deleterious when in compound heterozygosity with a missense *NPHS2* variant in trans, if occurring between exons 7 and 8 [86].

*PLCE1* OMIM 608414 encodes for phospholipase C epsilon 1, and it is one of the major causes of isolated DMS during childhood [87]. However, when *PLCE1* non-truncation mutations occur, they may cause adult FSGS as a degenerative defect more than as a result of a developmental defect [27, 88]. Also, *PLCE1* was recently identified as a regulator of podocyte migration and differentiation through Rho GTPase interaction [89].

*LAMB2* (OMIM 150325) encodes a GBM component, working beside COLA4 heterodimers. It is one of the most common causes of isolated CNS, but also causes a syndromic form of FSGS in the context of Pierson syndrome, characterized by CNS, microcoria, and neurodevelopmental disorders [90].

While *NPHS1*, *NPHS2*, *PLCE1*, and *LAMB2* are mostly associated with early-onset severe NS during the fetal period or first year of life, with rapid progression to KF, other recessive genes like *MYO1E* (OMIM 601479) are more likely associated with childhood-onset FSGS/SRNS and a later development of KF. Furthermore, *MYO1E* has been also associated with MCD biopsy findings [91]. As expected, recessive genetic causes are more frequently found in children, with a more severe and highly penetrant phenotype, while autosomal dominant genes are more frequently mutated in adults, with *WT1* as an exception, because it is associated with a broader age of onset range of disease [92].

*INF2* (OMIM 610982) is the most frequent autosomal dominant gene, responsible for 9–17% of adult familial FSGS, while *TRPC6* (OMIM 603652) and *ACTN4* (OMIM 604638) account for up to 12% and 3.5% of late-onset dominant familial FSGS, respectively [93–97]. *ACTN4* is a possible cause of sporadic cases, as well [98]. *INF2* encodes the inverted formin 2, involved in podocyte shape through actin cytoskeleton regulation and it is expressed in podocyte but also in heart, liver, and peripheral nerves, explaining the association with the Charcot-Marie-Tooth (CMT) neuropathy, in which FSGS is present in 75% of cases, as reported in the study from Boyer et al. [99].

*TRPC6* (OMIM 603652) encodes TRP cationic channel 6, involved in calcium traffic and representing one of the major components of the slit diaphragm [100]. It works closely with the cytoskeleton resulting in regulation of podocyte migration and motility [101].

*ACTN4* (OMIM 604368) encodes α-actinin-4 which provides foot processes adhesion to the GBM, leading to foot process effacement in both sporadic and familial FSGS [102, 103].

*TRIM8* (OMIM 606125) is an autosomal dominant gene recently identified in a very large cohort of pediatric individuals with SRNS/FSGS and in patients with epilepsy, with most of the pathogenic truncating mutations located in the last exon of the gene, very close to the C-terminal region [104].

Syndromic FSGS may occur in case of mutations in *WT1*, *PAX2*, *SMARCAL1*, *LMX1B*, *LAMB2*, and COQ10-related kidney nephropathies. *PAX2* (OMIM 167409) encodes a transcription factor important for brain, eye, and embryonic kidney development. *PAX2* has historically been associated with Papillo-renal syndrome, characterized by congenital abnormalities of the kidney and urinary tract (CAKUT), mostly renal hypoplasia and vesicoureteral reflux (VUR), and coloboma [105]. Since 2014, it has been identified as a cause of adult-onset familial FSGS, even without congenital abnormalities or extrarenal associated phenotypes [36]. Little is known about the molecular pathway leading *PAX2* to cause FSGS, but one hypothesis could be the regulation of *WT1* by *PAX2* [106], or a maladaptive response in case of *PAX2*-induced CAKUT with reduced nephron mass. Thus, *PAX2* probably represents one of the first examples of *phenocopy* in FSGS. *WT1* (OMIM 607102) is an autosomal dominant gene associated with the development of isolated Wilms tumor, isolated nephrotic proteinuria, or in the setting of syndromic conditions like Denys–Drash syndrome (DDS) and Frasier syndromes (FS), both including FSGS in association with sexual abnormalities [107].

*APOL1* OMIM 603743 is a common gene following recessive Mendelian trait, frequently mutated in a specific subpopulation. *APOL1* is an interesting gene with a high frequency of mutation in African Americans with sub-Saharan ancestry, leading to a three- to fourfold increased risk of developing FSGS and a twofold increased risk of developing KF. While *APOL1* variants confer protection from sleeping sickness, high-risk genotypes (G1-G1, G1-G2, G2-G2) increase the risk of developing glomerulosclerosis, as demonstrated by transgenic mice with podocyte-specific expression of *APOL1* G1/G2 alleles which develop proteinuria, foot process effacement, and FSGS. *APOL1* high-risk genotype is also associated with viral infections such as HIV, COVID-19, and malaria [108–111].

## Hidden phenocopies behind FSGS

### COL4A spectrum disorders

A large proportion of unknown CKD and KF may hide a genetic disease–causing defect [112].

A progressively increasing number of studies have disclosed how FSGS/SRNS may start from genetic mutations in genes far different from those classically defined as “podocyte-related.” FSGS may be difficult to differentiate from Alport syndrome (AS), based just upon pathology findings and symptoms, and it has been demonstrated by analysis of genetic insights that AS may often be mislabeled as FSGS [113, 114].

AS is an inherited glomerular disorder caused by pathogenic mutations of collagen alpha 4 genes (*COL4A3* (MIM: 203780; 104200; 620320), *COL4A4* (MIM 203780; 141200), *COL4A5* (MIM 301050). It is the most common glomerular inherited disorder. Hematuria, hearing loss, and progressive KF are the most typical symptoms related to AS. It has been estimated that in Europe, untreated patients affected by X-linked AS may rapidly evolve to progressive KF with a median age of 22 years [115], with males strongly affected and showing a more severe phenotype than females, in whom a less severe and variable phenotype is more common, due to X-chromosome inactivation (lyonizations) [116, 117].

Collagen IV represents the most abundant protein found in the GBM, and it strongly brings together podocytes and endothelial cells in the proper function of the glomerular filtration barrier. About 80% of AS patients may carry an X-linked mechanism of inheritance involving the *COL4A5* gene, with high penetrance of hematuria in males, showing the most severe phenotype. About 15% of patients with AS may show a recessive mode of inheritance due to mutations in *COL4A3* or *COL4A4*, both located on chromosome 2. However, a small portion of individuals, often underdiagnosed, and accounting for about 5% of AS patients, may show milder clinical manifestations with an autosomal dominant pattern of disease where just one mutated copy of *COL4A3*, *COL4A4* is identified; these patients are frequently defined as patients affected by thin basement membrane disease (TBMD) [116, 118–120]. Also, digenic inheritance has been reported [29, 121].

More than five thousand pathogenic variants have been recognized in *COL4A3*, *COL4A4*, and *COL4A5*, with 50% of missense variants affecting glycine residues, 20% of which variants are truncating nonsense mutations and frameshifts, while 15% respectively are large indels and deletions or variants altering the splicing mechanism [122].

Interestingly, *COL4A3*, *COL4A4*, and *COL4A5* pathogenic variants have been found in patients with persistent proteinuria or SRNS associated with FSGS, in both children and adult populations [123, 124]. Indeed, when large cohorts of CKD patients have been subjected to genotyping through WES or WGS, a high incidence of collagen IV pathogenic variants was found [125]. Interestingly, many recent reports and studies reveal that collagen IV genes are becoming the most common monogenic cause of FSGS in adults [126, 127].

So it is more common now to talk about COLA4-spectrum disorders, more than AS-related disease. Barua et al. performed WES in 193 patients with familial and sporadic forms of FSGS, using a gene panel of 109 genes related to FSGS, NS, CAKUT, and nephronophthisis. Pathogenic mutations in 28% of patients with a positive family history and 11% for sporadic cases were reported. Overall, the diagnostic yield for definitely pathogenic variants reached 11% of the total cohort, while 9% were likely pathogenic mutations. Interestingly, more than half (55%) of the pathogenic variants involved all the three collagen IV genes, *COL4A3*, *COL4A4*, and *COL4A5*, usually implicated in AS [128]. In another study from the Columbia University group, where one of the largest cohorts was sequenced, including 3315 patients affected by CKD, Groopmanan et al. identified monogenic disorders in 10% of the cohort, of those, about 100 patients, accounting for 30% of the diagnostic yield, showed mutations in *COL4A3*, *COL4A4*, or *COL4A5*. This study demonstrated that collagen IV variants were the second most frequent genetic disorders in the CKD cohort, after the 31% of *PKD1* and *PKD2* pathogenic variants. Only 35 out of 91 patients (38%) with diagnostic variants of collagen IV had a clinical diagnosis of AS or TBMD [129].

These findings demonstrate that collagen IV gene mutations represent one of the leading genetic causes of masked FSGS, often unrecognized, suggesting the importance of genetic screening in clinical practice. Genotype–phenotype correlation should be considered as a powerful tool to properly deliver tailored diagnoses, relative personalized treatment, and follow-up.

### Lysosome storage dysfunction

Lysosomal storage disorders can cause podocyte damage, mimicking histological features of FSGS. Alterations in genes encoding for lysosome proteins are responsible for Fabry disease, cystinosis, Nieman-Pick disease, and Tay-Sachs disease all characterized by kidney involvement [130].

Podocytes do not have the ability to proliferate; thus, intracellular homeostasis is important for their integrity. Lysosomes are essential organelles for the survival of podocytes, for their digestive and recycling properties [15].

Among lysosomal storage diseases, Fabry disease (FD) is an X-linked disorder, caused by mutation of the *GLA* (MIM 301500) gene, with defect of the enzymatic activity of the α-galactosidase enzyme (α-GalA), leading to abnormal and excessive deposition of neutral glycosphingolipids, including globotriaosylceramide (Gb3) in endothelial, epithelial, and smooth muscle cells. Progressive accumulation of glycosphingolipids causes clinical abnormalities of kidney, heart, skin, eye, brain, and peripheral nervous system. The accumulation of glycosphingolipids in renal lysosomes causes a progressive worsening of kidney function often resulting in KF [131].

In the early stages of FD, patients may show difficulties in concentrating urine, together with non-nephrotic proteinuria and modest hypertension, finally leading to impaired kidney function often resulting in KF in the third to fifth decades of life [132]. FD is therefore a multisystem and progressive disease.

FD may show histological features of FSGS. The morphologic alterations are determined by Gb3 deposits in all components of kidney parenchyma: glomerular, tubular, interstitial, and vascular. The deposits are observed in visceral podocytes earlier than in the Bowman’s capsule epithelium, in mesangial cells, in endothelial cells of glomeruli and peritubular capillaries, in the smooth muscle cells of arteries, in tubular cells, and most frequently in the distal tract. Interstitial cells are rarely involved. In advanced cases of disease, there are signs of segmental or global glomerulosclerosis, interstitial fibrosis, tubular atrophy, and arteriosclerosis [133]. The lysosomal deposits are lamellar electron dense structures (intercalated with electron-lucid lamellas), commonly termed “zebra bodies,” or “myelin figures” visible at the electron microscopy analysis of kidney biopsies. However, although electron microscopy is very useful to recognize Gb3 deposits associated with FD, they can be observed also in other conditions, such as silica nephropathy and pseudolipidosis, caused by the use of drugs such as amiodarone, chloroquine, and hydroxychloroquine.

Trimarchi et al. described the significant impact of electron microscopy in the specific differential diagnosis of FD in a patient initially classified as having FSGS by the analysis of kidney tissue only by light microscopy. They found lamellar electron dense lipids, as zebra bodies, under examination with electron microscopy in this 37-year-old patient initially treated with steroids as having FSGS for a long time. The correct diagnosis of FD allowed them to start the correct enzymatic replacement therapy [134]. The development of glomerular sclerosis in FD would seem to be mediated by an inflammatory state due to the deposition of Gb3 in the tissues. The increase of cytokines such as TGF-ß would therefore be responsible [135].

Data deriving from studies on the immune system of patients with FD are very interesting. Lymphocytes, monocytes, and granulocytes of patients with FD express more adhesion molecules than those in the healthy population [136]. Furthermore, Gb3 activates Toll-like receptor 4 (TLR4) that stimulates immune cells through Notch1 and the NF-κB transcription factors, with release of proinflammatory and profibrotic cytokines [137]. TGF-ß is crucial for fibrotic damage in response to chronic inflammation in FD, determining the synthesis of extracellular matrix in kidney cells via epithelial-to-mesenchymal transition. Indeed, deposition of Gb3 in glomerular cells is followed by FSGS until global glomerular sclerosis [138]. Studies of urinary proteomics revealed the presence of fibroblast growth factor 23, uromodulin, and podocalyxin in patients with FD, responsible for an inflammatory state and the activation of the fibrosis pathway in these patients. Enzyme replacement therapy can reduce the inflammatory state by reducing Gb3 deposits, only if administered in the early stages of FD. A late onset of enzyme replacement therapy is less effective on renal pathology, when fibrogenesis processes have already begun.

Another syndrome characterized by lysosomal anomalies is action myoclonus–renal failure syndrome (AMRF). It is an autosomal recessive progressive myoclonus epilepsy (PME) associated with kidney dysfunction, caused by loss-of-function mutations in the *SCARB2* (MIM 254900) gene encoding lysosomal integral membrane protein type 2 (LIMP2). This very rare syndrome appears in the second or third decade of life. LIMP2 traffics β-glucocerebrosidase to the lysosomal membrane. Mutations lead to glucosylceramide accumulation and neurologic symptoms including progressive action myoclonus, seizures, and ataxia [139]. Kidney involvement in AMRF consists of proteinuria that can evolve to NS, and even development of KF [140].

Badhwar et al. in 2004 described 15 cases with AMRF, all patients showing proteinuria, detected between age 9 and 30. The kidney biopsies performed in these patients showed collapsing FSGS. *SCARB2*/LIMP2 mutation also causes failure of endosomes containing reabsorbed proteins to fuse with lysosomes in the proximal tubular epithelial cells, with development of tubular proteinuria [141].

There are other lysosomal dysfunction diseases characterized by kidney impairment, mainly due to alteration of the proximal tubular compartment, with Fanconi syndrome, low molecular weight proteinuria, and even progressive KF. Cystinosis, Dent disease, and Lowe syndrome are due to genetic defects responsible for severe kidney damage. KF can be explained by the development of tubulointerstitial fibrosis [142].

Renal lipid dysregulation is furthermore one of the factors responsible for the development of diabetic nephropathy.

### Tubulointerstitial disease

Since the KDIGO consensus conference in 2015, different subclasses of autosomal dominant tubulointerstitial kidney disease (ADTKD) have been classified based on the genetic background [143].

Among these genes, *UMOD* (OMIM 191845) is a gene encoding uromodulin (also known as Tamm-Horsfall protein) that is the most abundant protein in normal urine. Uromodulin is essential in the regulation of ion transport, immunomodulation, protection against urinary tract infections, and prevention of the formation of kidney stones and oxidative stress [144, 145].

*UMOD* gene mutations are known to be related to ADTKD, also known as ADTKD-UMOD, which may slowly progress to CKD, leading to KF [146].

Gast et al. [147] analyzed patients with CKD stages 3–5, in order to identify patients with inherited kidney disease. They observed that ADTKD-UMOD was the most common genetic form of kidney disease after autosomal dominant polycystic kidney disease.

Moreover, Groopman et al. [129], conducting exome sequencing and diagnostic analysis in patients affected by CKD, identified 66 distinct monogenic disorders, and found that 3% were explained by mutations in *UMOD*, in a very large cohort of 3315 CKD patients [129]. Under a clinical profile, about 80% of patients affected by ADTKD-UMOD presented hyperuricemia that starts before the progressive loss of kidney function and is the main symptom of the disease. Additionally, gout and medullary renal cysts are sometimes present. ADTKD-UMOD is a difficult condition to diagnose, requiring a high clinical suspicion and confirmation by genetic testing. The urinary sediment is bland with absent to mild albuminuria or proteinuria and no hematuria. Patients with *UMOD* mutation usually develop KF between the third and sixth decade of life, whereas the onset of gout occurs between the ages of 3 and 51 years [147].

Renal pathology is usually unspecific, and patients affected by ADTKD-UMOD may be mislabeled as FSGS [22]. Electron microscopy may describe fibrillary intracellular deposits of uromodulin, stored within endoplasmic reticulum in tubular cells of Henle’s loop, explaining the frequently defective urine-concentrating process [148].

Thus, in patients with histological diagnosis of FSGS in whom an underlying secondary cause of FSGS is suspected, it is necessary to obtain a correct medical and family history for gout or kidney disease (FSGS of unclear etiology) and testing serum urate levels and urine analysis. In case of a strong clinical suspicion of ADTKD-UMOD, genetic tests are recommended to detect any mutations in *UMOD* gene.

*CLCN5* (OMIM 300008) is an X-linked recessive gene expressed in proximal tubules and collecting duct. It is responsible for a rare syndromic condition called Dent disease type 1 (Dent-1), characterized by hypercalciuria, nephrocalcinosis, kidney stone development, CKD, and progression to KF in which tubular proteinuria occurs. Sometimes proteinuria may reach nephrotic range values and it may be mistaken for a glomerular defect [149], and a glomerulosclerosis phenotype is possible [150]. The hypothesis is that *CLCN5* may cause FSGS and NS through regulation of podocyte trafficking, in addition to tubular dysfunction [151], so the effective molecular targets of *CLCN5* have not yet been fully clarified. Also, mutations in *OCRL* (OMIM 300535) may cause a severe tubular dysfunction called Lowe syndrome in the setting of Dent disease type 2 (Dent-2), characterized by ocular abnormalities, intellectual impairment, CKD, and rapid progression to KF, in which persistent proteinuria and FSGS have been described, as well [152]. Thus, *CLCN5* and *OCRL* should be taken into consideration as potential phenocopies of FSGS, in a genetic setting.

### Ciliopathy

Ciliopathy identifies a group of genetic disorders characterized by retinal degeneration, cerebral abnormalities, and kidney dysfunction and frequently presenting nephronophthisis (NPHP), a recessive condition frequently leading to CKD in young adults [43].

Many genes have been identified as disease-causing in NPHP [153]. However, three genes have been implicated in FSGS reports.

*TTC21B* (OMIM 612014) encodes for IFT139, an intra-flagellar transport-A component located at the primary cilium of young podocytes, while in adults in non-ciliated podocytes IFT139 is subjected to redistribution along the intracellular microtubule compartment. While *TTC21B* had been initially recognized as a potential genetic cause of NPHP (OMIM 613820), and short-rib thoracic dysplasia 4 with or without polydactyly it has also been reported as a possible genetic cause of glomerular compartment defects, in addition to tubulointerstitial alterations, manifesting FSGS [154–156].

*CC2D2A* (OMIM 612013) encodes a ciliary protein which works as a barrier to restrict protein flow between the ciliary membrane and plasma. Recently, a compound heterozygous missense mutation in *CC2D2A* has been reported in a girl affected by NPHP and FSGS [157].

*NPHP4* (OMIM 606966) is a recessive gene causing Senior-Loken syndrome 4 [158] and it has been identified in a single consanguineous family with segregation of proteinuria and kidney phenotype in multiple siblings, with a single patient undergoing kidney biopsy and diagnosed with FSGS [159]. The mechanism of disease causing FSGS through NPHP genes remains unexplained, but it is possibly a secondary adaptive response to nephron loss or podocyte cytoskeleton dysfunction in *TTC21B* mutations.

## Conclusions

About 10% of the population affected by CKD has a monogenic disorder [160, 161].

CKD is a complex disease, with different molecular mechanisms responsible for progressive kidney function decline. Patients affected by progressive CKD may show nonspecific histopathological features at kidney biopsy, such as a wide spectrum of glomerulosclerosis, interstitial fibrosis, and tubular atrophy that can be due to different pathogenic mechanisms. Thus, in a simplistic view, glomerulosclerosis may represent both a sign of progression of chronic inflammation and kidney injury, as well as a renal pathology hallmark in the diagnosis of FSGS, remaining an unspecific sign, detectable in different renal diseases.

It has been estimated that about 25% of dialyzed patients are classified as patients affected by KF of unknown origin. Thanks to the integration of DNA sequencing and genotyping approaches in kidney diseases, it has been demonstrated that a large proportion of patients with KF may remain unclassified, eventually hiding a genetic disease–causing defect [112].

Among these patients, FSGS, whose incidence is growing [3–5], represents a very heterogenous and complex disease. The recent updated KDIGO classification suggested the importance of identifying the underlying cause of primary, secondary, and genetic FSGS, required for personalized clinical management and treatment options.

So far, over 60 genes have been identified as monogenic causes of FSGS. FSGS and SRNS are frequently used synonymously due to the lack of immunosuppressive response especially in adults. Podocyte genes are commonly mutated in both familial and sporadic cases, but recent insights obtained from massive sequencing analysis on large cohorts of CKD patients have demonstrated that new patterns of injury need to be investigated as phenocopies in FSGS.

FSGS/SRNS management needs a new updated framework, which should consider an integrated approach between phenotype characterization, pathophysiology, and genetic testing to properly identify the correct causes of disease and to specifically drive treatment options, avoiding side effects and complications. Genetic versus non-genetic etiologies of SRNS and FSGS may have different prognosis, especially during childhood and in those resistant cases eventually planning a living donor transplant. Genetic testing is needed for familiar screening to determine donor eligibility status and to identify unsuitable potential familiar donors carrying one of the known genetic variants [22]. Thus, NGS should become a diagnostic standard.

Collagen IV genes including *COLA4A3*, *COL4A4*, and *COL4A5*, usually associated with hereditary forms of Alport syndrome, represent the emerging most frequent cause of FSGS in patients with otherwise unknown CKD or KF. Moreover, the growing interest in rare complex diseases, such as Fabry disease, has revealed that FSGS may hide mutations in the *GLA* gene leading to lysosomal dysfunction, manifesting glomerulosclerosis features at the kidney level. Even if glomerular and tubulointerstitial compartments seem to be separate sites of damage, some of the genes regulating tubular homeostasis and cilia structure may show a sort of dualism. *UMOD*, *CLCN5*, *OCRL*, *NPHP4*, and *TTC21B* may cause tubulointerstitial diseases such as ADTKD, NPHP, or Dent disease, but they are now included in the genetic panels for genetic screening of patients affected by FSGS, as they can phenocopy it. Many other new genes classically involved in syndromic/non-syndromic disorders, have been identified in sequencing analysis of patients showing FSGS phenotype.

In conclusion, new insights into FSGS heterogeneity represent an opportunity, because it moves the attention from podocytes to other areas of interest, discovering new potential triggers of damage, manifesting with proteinuria and glomerular scars. The incomplete penetrance and pleiotropic expression of FSGS/SRNS require a broader genetic analysis in order to provide a tailored and targeted diagnosis and for treatment selection.

Glomerular scars are not a specific and distinctive sign of FSGS; however, they represent the hallmark in the diagnosis of this proteinuric disease. In addition, FSGS classification has been subjected to rearrangements, and new monogenic causes of FSGS are discovered on a monthly basis. When we look at a kidney biopsy specimen through the lens of a light microscope, we cannot understand what is hidden behind glomerular scars, but we can just describe the captured features. An integrated approach that includes patient “phenotyping,” renal pathology, clinical reports, and sequencing analysis is now mandatory to interpret the data and to offer the better diagnosis and management to patients affected by kidney diseases, in the era of precision medicine.
